# Fomite Transmission of SARS-CoV-2 and Its Contributing Factors

**DOI:** 10.3390/pathogens12030364

**Published:** 2023-02-22

**Authors:** Taeyong Kwon, Nikolaus Osterrieder, Natasha N. Gaudreault, Juergen A. Richt

**Affiliations:** 1 Department of Diagnostic Medicine/Pathobiology, College of Veterinary Medicine, Kansas State University, Manhattan, KS 66506, USA; 2 Institut für Virologie, Freie Universität Berlin, Robert-von-Ostertag-Str. 7-13, 14163 Berlin, Germany

The emergence of severe acute respiratory syndrome coronavirus 2 (SARS-CoV-2) has drastically changed our lives, from our personal freedoms and habits to public health and socioeconomics. Since its inter-species and zoonotic transmission from bats to humans via a yet unknown intermediate host, SARS-CoV-2 has circulated in the human population and resulted in almost 7 million recorded deaths worldwide (February 2023). The transmission of SARS-CoV-2 occurs by inhalation of droplets and aerosols from infected individuals and animals, and, to some extent, by exposure to fomites.. Direct transmission is by far the most common route of the human-to-human transmission of SARS-CoV-2. Aerosols are generated by exhalation of very fine droplets (less than ≤5 μm in size), which can remain suspended in the air for an extended period of time, allowing the virus to disperse from infected individuals and then infect others. Due to the small size of aerosols, the virus can reach the lower respiratory tract, which enables the virus to initiate infection and replicate in the lungs, causing severe damage to tissues and respiratory disease. In contrast, larger droplets or infectious biological fluids from infected humans and animals usually contaminate nearby surfaces and environments, and thus increase the risk for fomite transmission. In reality, fomite transmission seems to be a relatively rare event as it requires a multi-step process, which involves environmental contamination, virus survival outside of the host, and the mechanical transfer of more than the minimal infectious dose of SARS-CoV-2 to susceptible individuals.

Van Doremalen et al. [1] described SARS-CoV-2 stability on surfaces and in aerosols. These authors found that the stability of SARS-CoV-2 is very similar to that of its cousin, SARS-CoV, thus providing the first evidence of potential risks that contaminated surfaces and aerosols are posing in SARS-CoV-2 transmission. Subsequent studies identified the role of environmental factors on SARS-CoV-2 stability [2,3]. Kwon et al. [2] demonstrated remarkable SARS-CoV-2 stability on twelve different surfaces, which represent common materials and personal protective equipment. The contaminated surfaces were incubated under indoor, summer, spring/fall, and winter conditions that were chosen to mimic different climate conditions of temperate zones. The stability of SARS-CoV-2 on surfaces was highly dependent on climatic conditions. The longest survival was observed under winter conditions, followed by spring/fall and summer conditions. In addition, the role of simulated sunlight on SARS-CoV-2 stability was described by Ratnesar-Shumate et al. [3]. The exposure to UVB light, which is present in sunlight next to UVA light, accelerated the inactivation of SARS-CoV-2 on a stainless-steel surface in an energy-dependent fashion. These data suggest that the potential risk of indirect transmission through contaminated surfaces may vary significantly between indoor and outdoor environments.

Substrates in the environment also contribute to SARS-CoV-2 stability. SARS-CoV-2 is mainly excreted through respiratory droplets, in which nasal mucus, sputum, and saliva are major components. In addition, SARS-CoV-2 has been isolated or detected in tears, urine, feces, blood, breast milk, and semen from infected humans. These findings have prompted the investigation of SARS-CoV-2 stability in human biological fluids and the potential of fomite transmission through bodily secretions and excretions that mimic real-world situations. Kwon et al. [4] spiked nine clinically relevant biological fluids with SARS-CoV-2 and evaluated virus stability. The virus was stable for up to 21 days in nasal mucus, sputum, saliva, tears, urine, blood, and semen, whereas the virus was only stable for up to 24 h in feces and breast milk. In addition, SARS-CoV-2 can enter the wastewater system via contaminated feces and urine, motivating investigations on SARS-CoV-2 stability in water and wastewater [5]. SARS-CoV-2 remained infectious in wastewater for at least 7 days, but heat treatment rapidly inactivated SARS-CoV-2 infectivity in wastewater. These findings illustrate the potential risks of infectious human biological fluids and their byproducts in SARS-CoV-2 transmission.

Furthermore, the particular SARS-CoV-2 strain could be a factor that determines virus survival outside of the host. Since its emergence, SARS-CoV-2 has continued to accumulate mutations throughout its genome, which lead to altered phenotypes of pathogenicity, immunogenicity, and transmissibility. Since single or combinations of mutations could have an effect on the environmental stability of SARS-CoV-2, a growing body of work has been generated to determine the environmental stability of SARS-CoV-2 variants of concern (VOCs). In this context, our laboratory showed that the Wuhan-like ancestral strain is more stable than the alpha, beta, and omicron VOCs in various human biological fluids [6]. It is hypothesized that amino acid substitutions in viral structural proteins might be responsible for the reduced stability of SARS-CoV-2 VOCs. These results indicate that the environmental stability of novel variants needs to be tested to elucidate the potential risk in fomite transmission.

In addition to virus stability, the efficiency of the mechanical transfer of the virus from surfaces plays a role in fomite transmission. In most scenarios, the hand and fingers act as an intermediate bridge that facilitates the transfer of the virus from contaminated surfaces to target cells in the human upper respiratory tract. Todt et al. [7] developed a new touch-transfer assay to determine the efficiency of virus transfer to fingertips. Viruses such as bovine coronavirus or SARS-CoV-2 were added to surfaces and touched by printing or rubbing using fingertips or an artificial skin fabric, respectively. The transfer efficiency varied depending on the virus inoculum, surface materials, and touch techniques, but the results suggest that infectious viruses could be transferred onto fingertips, thus emphasizing the potential role of fingers and hands in the mechanical transfer of SARS-CoV-2.

The minimum infectious dose of pathogens is the smallest quantity of infectious particles that produce an infection. Host, pathogen and environmental factors can all influence the minimum dose for infection. Unlike the situation of pathogens entering the organism via the enteric route, in which viruses or bacteria encounter extremely acidic environments in the stomach and a variety of enzymes in the gastrointestinal tract, the minimum infectious dose of respiratory pathogens is generally considered low. This is due to the relatively direct exposure of susceptible respiratory cells in the upper respiratory tract to virus-containing materials and relatively mild physicochemical and physiological conditions in this locality, which enable pathogens to initiate infection more directly and easily. In animal models, intranasal inoculation with ten 50% tissue culture infectious dose (10 TCID_50_) of SARS-CoV-2 was sufficient to induce a productive infection in 100% of Syrian hamsters [8]. More importantly, Billingley et al. [9] performed a human SARS-CoV-2 challenge study in which they evaluated the kinetics of virus shedding and disease progress. Surprisingly, intranasal inoculation with 10 TCID_50_ of the ancestral strain of SARS-CoV-2 resulted in 53% (18/34) of the healthy individuals becoming infected. Given that more transmissible SARS-CoV-2 strains are circulating in immunologically and demographically diverse human populations, the minimal infectious dose in real-world situations could potentially be lower than under experimental conditions. This might have significant implications for the potential role of fomite transmission since the infectivity of excreted viruses present in the environment is rather low and naturally decreases over time. Consequently, a low minimal infectious dose for SARS-CoV-2 in humans presents a higher risk for the fomite transmission of this pathogen.

There is still a knowledge gap with regard to the potential risk of fomite transmission in SARS-CoV-2 ecology. SARS-CoV-2 is a zoonotic virus which is capable of crossing species barriers and has been detected in a wide range of animal species [10]. Nonetheless, the stability of SARS-CoV-2 in animal biological fluids and the potential risk from the environmental contamination by animal secretions and excretions remain unclear. In particular, fomite transmission after introduction of SARS-CoV-2 into the environment may play a crucial role in wildlife infections because close physical interaction between humans and wildlife, and even among wild animals, is clearly less common than close interaction between humans. Therefore, it is critical to determine the environmental stability of newly emerging strains of SARS-CoV-2 in animal biological fluids in order to mitigate and prevent fomite transmission of SARS-CoV-2.

The world is still battling SARS-CoV-2, and we live with the certainty that this virus is increasing its fitness with respect to evading immune defenses and adapting to new environments. In addition to boosting our immune response and developing efficient antiviral therapeutics, breaking the transmission chains of SARS-CoV-2 is an effective countermeasure to mitigate the public health impact and the socio-economic losses associated with this pathogen. Specifically, we can relatively easy prevent fomite transmission by disinfecting surfaces and by cleaning hands regularly and thoroughly.
